# Post-Translational Modifications of ATG4B in the Regulation of Autophagy

**DOI:** 10.3390/cells11081330

**Published:** 2022-04-13

**Authors:** Na Yeon Park, Doo Sin Jo, Dong-Hyung Cho

**Affiliations:** BK21 FOUR KNU Creative BioResearch Group, School of Life Sciences, Kyungpook National University, Daegu 41566, Korea; yeonie5613@gmail.com (N.Y.P.); doosinjo@gmail.com (D.S.J.)

**Keywords:** ATG4, autophagy, post-translational modification

## Abstract

Autophagy plays a key role in eliminating and recycling cellular components in response to stress, including starvation. Dysregulation of autophagy is observed in various diseases, including neurodegenerative diseases, cancer, and diabetes. Autophagy is tightly regulated by autophagy-related (ATG) proteins. Autophagy-related 4 (ATG4) is the sole cysteine protease, and four homologs (ATG4A–D) have been identified in mammals. These proteins have two domains: catalytic and short fingers. ATG4 facilitates autophagy by promoting autophagosome maturation through reversible lipidation and delipidation of seven autophagy-related 8 (ATG8) homologs, including microtubule-associated protein 1-light chain 3 (LC3) and GABA type A receptor-associated protein (GABARAP). Each ATG4 homolog shows a preference for a specific ATG8 homolog. Post-translational modifications of ATG4, including phosphorylation/dephosphorylation, *O*-GlcNAcylation, oxidation, S-nitrosylation, ubiquitination, and proteolytic cleavage, regulate its activity and ATG8 processing, thus modulating its autophagic activity. We reviewed recent advances in our understanding of the effect of post-translational modification on the regulation, activity, and function of ATG4, the main protease that controls autophagy.

## 1. Introduction

**Figure 1:**
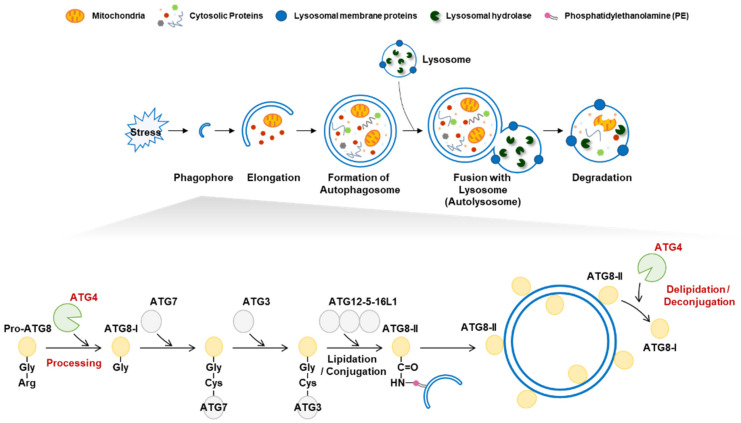
Autophagy activation and the role of autophagy-related 4 (ATG4). (Top) Autophagy is activated in response to various stress stimuli. A phagophore elongates into an autophagosome to engulf intracellular targets, including damaged organelles and aggregated proteins. Mature autophagosomes fuse with lysosomes to form autolysosomes that contain various hydrolytic enzymes that degrade target components. (Bottom) The role of ATG4 in lipidation and delipidation. The C-terminal extended preform of pro-ATG8 is cleaved by ATG4 cysteine protease to generate the ATG8-I form harboring an exposed C-terminal glycine residue. Two ubiquitin-like steps involving ATG7, ATG3, and the ATG12-5-16L1 complex facilitate conjugation of PE to ATG8-I, resulting in the formation of ATG8-II, which is tightly bound to the outer autophagosome membrane. ATG8-II can be further cleaved by ATG4 to release and recycle free ATG8-I.

Macroautophagy (autophagy) is a highly conserved catabolic process that maintains cellular homeostasis [1]. Autophagy plays an essential role in eliminating and recycling cellular components in response to various stresses [2]. Autophagy is initiated by the generation of a phagophore membrane, which elongates to form an autophagosome [2,3]. Mature autophagosomes fuse with lysosomes, and autophagic cargo is degraded by lysosomal hydrolases (Figure 1) [2]. Autophagy is tightly regulated by autophagy-related (ATG) proteins, and to date, approximately 40 genes encoding ATG proteins have been identified in yeast [4]. Autophagic dysregulation is observed under various disease conditions, including neurodegenerative diseases, cancer, and diabetes [5]. Understanding the regulatory mechanisms underlying autophagy is therefore critical.

Autophagy-related 8 (ATG8) is a ubiquitin-like protein that is widely used as an autophagosome marker [6,7]. In mammals, ATG8 has seven homologs: microtubule-associated protein 1-light chain 3A (LC3A), LC3B, LC3B2, LC3C, GABA type A receptor-associated protein (GABARAP), GABARAPL1, and GABARAPL2 [6]. ATG8 plays an essential role in autophagosome formation and phagophore elongation [7]. During autophagosome formation, ATG8 is modified via lipidation and delipidation by the cysteine protease ATG4 [8]. ATG8 lipidation is crucial for the initiation and elongation of mature autophagosomes [9]. To conjugate ATG8 to phosphatidylethanolamine (PE), full-length ATG8 (pro-ATG8) is cleaved by ATG4, exposing a glycine residue [10]. The cleaved ATG8 (ATG8-I) is then conjugated to PE on the autophagosome membrane to form ATG8-PE (ATG8-II) via two ubiquitin-like conjugation reactions [11]. In the first reaction, ATG7, ATG3, and the ATG5-ATG12-ATG6 complex elongate pro-autophagosomal membranes. The second process generates ATG8-II, which mediates membrane tethering and hemifusion. In addition, ATG4 cleaves ATG8-PE into delipidated ATG8 (ATG8-I) [12]. The deconjugation of ATG8-PE occurs on the autophagosome’s outer membrane, allowing the recycling of ATG8-I for membrane elongation [12,13].

Post-translational protein modifications such as adding covalent functional groups, proteolytically cleaving regulatory subunits, or degrading proteins increase the functional diversity of the proteome. Autophagy is modulated by post-translational modifications, including phosphorylation, *O*-GlcNAcylation, and redox modifications [14]. Several post-translational modifications of ATG4 homologs regulate their activity and the processing of ATG8 homologs, leading to the modulation of autophagy. In this review, we focused on recent advances in our understanding of the effects of post-translational modifications on the regulation, activity, and function of ATG4B, the main protease that controls autophagy.

## 2. Structure and Function of ATG4

Autophagy-related 4 (ATG4) is the only ATG that displays cysteine protease activity [8]. Four ATG4 homologs are found in mammals: ATG4A, ATG4B, ATG4C, and ATG4D. ATG4 is composed of 393–474 amino acids and has two domains: short fingers and catalytic [15,16]. The short fingers domain is unique to ATG4 and is located within the catalytic domain [15,16]. The catalytic domain is conserved within papain-family cysteine proteases, and the catalytic active site includes Cys-74, Asp-278, and His-280 of ATG4B, a canonical triad of cysteine proteases [10,15]. Mutations in these residues block proteolytic activity [8]. In particular, the Cys-74 residue is masked by an inhibitory loop (residues 259–262), but a conformational change occurs after binding to substrates such as ATG8/LC3 [17]. ATG4 has an LC3-interacting region (LIR) motif at its C-terminus that is essential for binding and processing LC3 (Figure 1) [18]. ATG4C and ATG4D have the canonical DEVD (aspartic acid, glutamic acid, valine, and aspartic acid) sequence that is recognized by caspase, as well as latent mitochondrial-targeting motifs near caspase cleavage sites [19,20]. Catalytic sites are located at the N-termini of ATG4C and ATG4D [21]. Each ATG4 isoform has a preference for a specific ATG8 homolog. ATG4A prefers GATE-16, ATG4B prefers LC3B processing over other ATG8s [22], and both ATG4C and ATG4D target GABARAP [23]. Although the contribution of individual ATG4 homologs to autophagy remains to be determined, ATG4-deficient mice show reduced autophagic flux [24,25,26]. Notably, ATG4B depletion dramatically reduces autophagy and LC3/GABARAP processing, although other ATG4 homologs are sufficient for lipidation and autophagosome localization of GABARAP isoforms [23]. Therefore, the redundancy of ATG4 isoforms is compensatory during autophagy, resulting in the viability of ATG4-deficient mice [23].

Dysfunctional autophagy is associated with several pathological conditions [5,13]. ATG4 dysregulation results in abnormal autophagy during disease development or progression [5]. For example, ATG4 expression is upregulated in various cancers, including colorectal and gastric cancers [27,28]. The upregulation of ATG4A and ATG4B increases cancer cell proliferation, migration, and invasion in vitro and metastasis in vivo [28,29,30]. Conversely, ATG4D is downregulated in uterine fibroids, and its depletion increases the proliferation of normal human myometrial cells and production of the extracellular matrix [31]. Thus, inhibiting ATG4 may be a potential strategy for anticancer therapy. Various ATG4 inhibitors such as tioconazole [32], UAMC-2526 [33], NSC185058 [34,35], S130 [36], and flubendazole [37] reduce autophagic flux and increase cancer cell death [8]. Thus, the regulatory mechanisms underlying ATG4 activity in various pathological conditions require further investigation.

## 3. Post-Translational Modifications of ATG4

**Figure 2:**
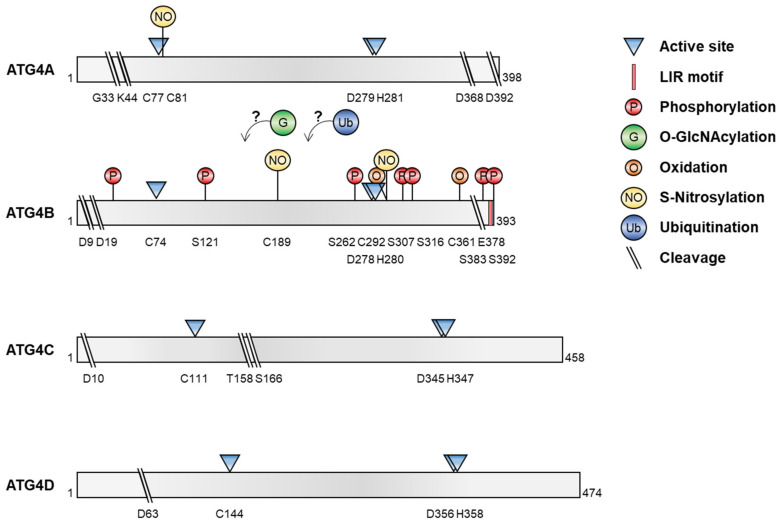
Post-translational modification of ATG4. The diagrams show the various post-translational modifications of ATG4 and the residues targeted during modification.

Recent reports have shown that several post-translational modifications of ATG4, including phosphorylation/dephosphorylation, *O*-GlcNAcylation, oxidation, S-nitrosylation, ubiquitination, and proteolytic cleavage, regulate its proteolytic activity and ATG8 processing, leading to the modulation of autophagy under various conditions (Figure 2 and Table 1).

### 3.1. Phosphorylation

Protein phosphorylation, the most common post-translational modification, involves the reversible addition of phosphoryl groups to serine, threonine, and tyrosine residues [49]. Phosphorylation influences various cellular functions, including cell survival, death, and autophagy [50]. ATG4B has several target residues that can be phosphorylated and several large-scale phosphoproteomic studies have suggested that ATG4B is phosphorylated at Ser-34 [51], Ser-383, and Ser-392 [52,53,54]. ATG4B is phosphorylated at the C-terminal Ser-383 and Ser-392 in response to starvation, enhancing its hydrolase activity [40]. Phosphorylation of ATG4B at these residues increases the proteolytic cleavage of LC3. In addition, reconstitution of phospho-defective ATG4B mutants in ATG4B knockout MEF decreases LC3 delipidation by ATG4B and autophagic flux [40].

#### 3.1.1. ATG4B Phosphorylation by MST4

ATG4B is post-translationally modified by mammalian sterile 20-like kinase-26 (STK26)/MST4 [35]. Proteomic analysis of patient-derived glioma stem-cell-like cells (GSCs), with and without exogenous MST4 expression, showed that ATG4B is highly phosphorylated at Ser-383 in GSCs expressing MST4. MST4 directly phosphorylates ATG4B at Ser-383 [40]. Furthermore, prediction analysis suggests that the MST4 consensus phosphorylation site is highly conserved across species [55]. In silico analysis showed that the phosphorylation of ATG4 by MST4 is specific to ATG4B. Radiation, which upregulates MST4 expression, increases autophagic activity by upregulating phosphorylated-ATG4B (Ser-383). Conversely, inhibiting MST4 in glioblastoma (GBM) cells decreased both autophagy and tumorigenicity. These effects were reversed by the reconstitution of either wild-type or phosphomimetic mutants of ATG4B. Therefore, the MST4-ATG4B signaling axis contributes to GBM autophagy and malignancy [35].

#### 3.1.2. Phosphorylation by AKT1 and AKT2

ATG4B in hepatocellular carcinoma (HCC) cells is also phosphorylated by AKT Serin/Threonine Kinase 1 (AKT1) [41]. The AKT family of proteins regulates autophagosome formation and autophagy [56]. ATG4B has a potential role in tumor progression, but the effect of ATG4B phosphorylation remains unclear. Phosphorylation prediction analysis indicated that ATG4B has a putative phosphorylation motif (_31_RKYS_34_) for AKT1 at its N-terminus and identified the Ser-34 residue of ATG4B as a target in HCC cells. ATG4B phosphorylation by AKT1 had little effect on basal autophagic flux, although it enhanced the Warburg effect in HCC cells. Cells expressing wild-type ATG4B or a phospho-defective ATG4B mutant (S34A) with AKT1 had different levels of L-lactate, the main product of the Warburg effect. L-lactate was produced in wild-type ATG4B-expressing cells in greater quantities than in phospho-defective ATG4B cells. Overexpression of ATG4B phosphorylated on Ser-34 by AKT1 results in the inhibition of mitochondrial function via a decrease in F_1_F_0_-ATP synthase activity and an increase in mitochondrial reactive oxygen species (ROS). Therefore, phosphorylation of ATG4B at residue Ser-34 plays a noncanonical role under pathological conditions by regulating metabolic reprogramming [41].

AKT1 directly phosphorylates ATG4B at Ser-34. However, whether this phosphorylation increases or decreases ATG4B activity remains unknown. Pengo et al. showed that AKT2 is a novel gene that activates ATG4B [42]. High-throughput screening to identify kinases and phosphatases using a luciferase reporter assay targeting ATG4B activity revealed that AKT2 overexpression increased the luciferase signal and enhanced ATG4B activity. Furthermore, an in vitro kinase assay also predicted that both Ser-121 and Ser-262 of ATG4B could be phosphorylated by AKT2. Therefore, ATG4B may be phosphorylated at multiple residues by both AKT1 and AKT2 to regulate ATG4B activity [41,42].

#### 3.1.3. Phosphorylation by PFKP

The Ser-34 residue in ATG4B can be phosphorylated by phosphofructokinase 1 platelet isoform (PFKP), a rate-limiting canonical metabolic enzyme in the glycolytic pathway [43]. High-fidelity technology combined with tandem affinity purification and mass spectrometry identified PFKP as an ATG4B-interacting protein. PFKP, as a canonical metabolic kinase, interacts with ATG4B, and this interaction is enhanced by amino acid deprivation. Liquid chromatography–mass spectrometry (LC–MS) analysis of Flag-tagged ATG4B immunoprecipitants identified ATG4B phosphorylation at Ser-34 and PFKP at Ser-386. These reactions enhanced ATG4B activity and SQSTM1/p62 degradation under amino acid deprivation conditions. In addition, PFKP phosphorylation at Ser-386 was important for ATG4B Ser-34 phosphorylation and autophagy in HEK293T cells. Taken together, these results indicate that phosphorylated PFKP regulates ATG4B activity under amino acid-deficient conditions [43].

#### 3.1.4. Phosphorylation by Atg1/ULK1

In yeast, Atg1 kinase is an Atg protein that participates in phagophore formation. At least seven putative Atg1 phosphorylation sequences are conserved in Atg4. Atg4 is an Atg1 substrate and was phosphorylated at Ser-307 in an in vitro assay. The phosphorylation of Atg4 at Ser-307 inhibits interactions with Atg8 and cleavage activity, particularly at sites of autophagosome biogenesis. The Atg8-PE pool is critical for autophagosome formation and is partially protected by Atg4′s phosphorylation by Atg1. However, newly synthesized Atg8 is constitutively processed by Atg4 [57].

ULK1 also mediates ATG4B phosphorylation in mammalian cells [44]. A proteomic study of autophagy protein networks showed that ATG4B binds directly to ULK1 and ATG101 [58]. Exogenous expression of a catalytically active ULK1 mutant decreased the proteolytic activity of ATG4B, whereas a catalytically inactive mutant increased ATG4B function under nutrient deprivation, as demonstrated by a luciferase-based assay. Ser-316 of ATG4B is a putative ULK1 target, and the surrounding sequences are correlated with the ULK1 consensus motif [59,60]. An in vitro phosphorylation assay confirmed that ATG4B is phosphorylated by ULK1 at Ser-316. This phosphorylation and the phosphomimetic mutant of ATG4B inhibit interactions with LC3 and the processing ability of ATG4B.

Thus, several kinases bind to and phosphorylate ATG4B; however, the effects of phosphorylation on ATG4B activity are not fully understood. These findings point to the complex regulation of ATG4B activity by kinases and indicate that such phospho-regulation may be dependent on different kinases under different stress conditions [57].

### 3.2. ATG4 Dephosphorylation

Dephosphorylation is the removal of phosphoryl groups by phosphatases. Dephosphorylation and phosphorylation activate and deactivate proteins by detaching or attaching phosphate esters and anhydrides, respectively. Thus, phosphorylation–dephosphorylation cycles are common post-translational modifications. This process involves the reversible addition of phosphoryl groups to amine moieties on serine/threonine and tyrosine residues. Atg1/ULK1 negatively regulates ATG4B activity in yeast and mammalian cells [44,57]. An unknown phosphatase likely participates in the reactivation of ATG4B, which is inhibited by ULK1-mediated phosphorylation. A cDNA phosphatase library was screened using a cell-based ATG4B luciferase assay [44]. A newly identified protein, phosphatase 2A (PP2A), decreased the phosphorylation of ATG4B at Ser-316, thus increasing the ATG4B-mediated processing of LC3. A chemical inhibitor of PP2A enhanced ATG4B Ser-316 phosphorylation. Further investigations are needed to determine the regulation of phosphatase early on in autophagy. However, ATG4B’s cellular activity is regulated in a phosphorylation-dependent manner by ULK1 and phosphatase PP2A [44].

### 3.3. O-GlcNAcylation of ATG4

*O*-linked-*N*-acetylglucosaminylation (*O*-GlcNAcylation) is a glycosylation process that attaches a monosaccharide, *O*-GlcNAc, to serine or threonine residues [61,62], suggesting the possibility of a dynamic and extensive crosstalk between *O*-GlcNAcylation and phosphorylation. *O*-GlcNAcylation influences various cellular processes, including nutrient sensing, metabolism, and transcription [63]. *O*-GlcNAcylation is regulated by *O*-GlcNAc transferase (OGT) and is reversed by *O*-GlcNAcase (OGA) [61,63]. Dysregulation of *O*-GlcNAcylation is associated with several illnesses, including neurodegenerative diseases, diabetes, and cancer, all of which are also associated with autophagy disorders [63]. We previously showed that *O*-GlcNAcylation plays a role in sensing nutrients and stress conditions under which autophagy is initiated [64,65]. Treatment with PugNAc, an OGA inhibitor, induced GFP-LC3 puncta and autophagic flux [45]. Bimolecular fluorescence complementation and immunoprecipitation (IP) assays showed that ATG4B and OGT interact directly. Furthermore, *O*-GlcNAcylation of ATG4B was elevated following treatment with PugNAc, as well as under low-glucose conditions. A luciferase assay showed that *O*-GlcNAcylation increased LC3 cleavage activity. Specific target residues for ATG4B *O*-GlcNAcylation have not been identified, although mass spectrometry analyses showed several possible target residues, suggesting that ATG4B is *O*-GlcNAcylated at multiple residues following autophagic activation [45]

### 3.4. ATG4 Oxidation

ROS are signaling molecules involved in growth, differentiation, adhesion, and autophagy. Both free radicals and ROS induce oxidative post-translational modification (Ox-PTM), including protein hydroperoxides and hydroxylation of aromatic groups and aliphatic amino acid chains [66]. Ox-PTM can affect protein structure and function [67]. In response to starvation, ROS, particularly H_2_O_2_, are generated in a partial class III PI3K-dependent manner [38]. Various oxidative stresses induce autophagy, whereas antioxidants reduce autophagosome formation and protein degradation. In addition, during starvation, ATG4 is oxidized by H_2_O_2_ at a cysteine residue near the ATG4 proteolytic residue. Of the 12 cysteine residues in ATG4A, Cys-81 is an oxidative target for the redox regulation of ATG4A [38]. ATG4B is also a target for redox modification [38]. ATG4B activity was decreased by H_2_O_2_ treatment and restored by N-acetyl-L-cysteine treatment in an in vitro ATG4B proteolytic assay, suggesting that ATG4B is reversibly redox-regulated [46]. To identify the Ox-PTM site, 13 cysteine residues were mutated using site-directed mutagenesis. None of the mutants showed a direct impact on ATG4B activity in an in vitro cleavage assay. However, the double mutant C292S/C361S efficiently reduced ATG4B oxidation, indicating that these residues mediate Ox-PTM. Therefore, these cysteine residues may be required for generating oxidized ATG4B, via the formation of a disulfide bond, in response to oxidative stress. Furthermore, oxidation at Cys-292 and Cys-361 may lead to the generation of oligomers that reduce ATG4B activity. The C292A/C361A double mutant induced autophagic flux and reduced the sensitivity to oxidation under normal growth conditions. ATG4B is thus modified by the oxidation of Cys-292 and Cys-361, leading to the regulation of autophagy [46].

### 3.5. S-nitrosylation of ATG4

S-nitrosylation is a PTM that reversibly adds a nitric oxide (NO) group to cysteine residues to generate S-nitrosothiols (SNOs) in response to redox-mediated reactions [66,68]. S-nitrosylation controls the function of target proteins by affecting their activities (activation and inhibition), subcellular localization/distribution, and interaction with partners [68]. S-nitrosylation is a reversible process mediated by reducing compounds such as glutathione (GSH) and thioredoxins (Trx). NO also affects autophagy under various stress conditions [69]. ATG4B is modified via S-nitrosylation, resulting in the impairment of autophagy and further neurotoxicity in response to hyperglycemia [47]. Autophagy is downregulated in the hippocampi of animals with spontaneous type 2 diabetes under hyperglycemic conditions. Basal autophagy protects against high glucose-induced neurotoxicity. However, high glucose and NO-induced high glucose levels reduce autophagosome formation and inhibit autophagic flux. LC–MS-based proteomic analysis showed that residues Cys-189 and Cys-292 of ATG4B in the hippocampus of diabetic animal models are S-nitrosylated in response to NO, resulting in the inhibition of ATG4B′s ability to process ATG8 family precursors and deconjugate ATG8 to PE. The ATG8 family member, GABARAPL1 precursor, is influenced by ATG4B S-nitrosylation. Therefore, ATG4B S-nitrosylation-induced autophagic inhibition increases neurotoxicity and hyperglycemia [47].

### 3.6. Ubiquitination of ATG4

Ubiquitination is an enzymatic post-translational modification in which a small ubiquitin protein (8.5 kDa) is attached to substrate proteins. Ubiquitination affects proteins in various ways, including degradation in the proteasome system, alterations in cellular location-affecting activity, and promotion or prevention of protein interactions [66,70]. Ubiquitination proceeds sequentially through three steps: activation of ubiquitin by ubiquitin-activating enzymes (E1), conjugation by ubiquitin-conjugating enzymes (E2), and ligation by ubiquitin ligases (E3) [70]. Two protein conjugation systems similar to those involved in protein ubiquitination are required for autophagic vesicles [71]. ATG7 acts as an E1-like activating enzyme that binds ATG8/LC3 or ATG12. Activated ATG8 and ATG12 are then transferred to the E2-like conjugation enzymes, ATG3 and ATG10, respectively. Finally, ATG8-PE and the ATG12-ATG5 conjugate are formed. The conjugate binds to ATG16L1, forming a trimeric complex that acts as an E3-like enzyme for the ATG8-PE conjugate. Using this ubiquitin-like system, ATG8 is lipidated with PE on the phagophore. In addition, core autophagy regulators such as ULK1 are modified via reversible ubiquitination, controlling autophagy [70]. Screening cDNA libraries using a yeast-based functional assay identified RNF5, an 18-kDa RING finger E3 ligase, as a candidate regulator of ATG4B inhibition [48]. RNF5 affects ER stress, innate immunity, and bacterial infection and influences autophagy. Furthermore, ATG4B interacts with RNF5 under basal conditions, while the interaction between ATG4B and RNF5 decreases during starvation, implicating the negative regulation of autophagy by RNF5 under normal conditions. Additionally, RNF5 depletion induces the interaction between ATG4B and LC3, whereas its overexpression decreases binding. RNF5 mediates the polyubiquitination of ATG4B, whereas the RING mutant of RNF5 fails to induce ubiquitination. ATG4B expression was higher in RNF5-/- MEFs and LC3 processing increased in RNF5-knockdown cells. Moreover, the loss of RNF5 leads to increased basal levels of autophagy and autophagosome formation by enhancing ATG4B. RNF5 inhibition in *Caenorhabditis elegans* increased the levels of LLG-1/LC3:GFP puncta. Infection with group A *Streptococcus* was lower in RNF5-/- mice because autophagosome formation increased and mediated the clearance of bacteria by RNF5-/- macrophages. Overall, ATG4B is negatively regulated by RNF5 in a ubiquitin-dependent manner [48].

### 3.7. Proteolytic Cleavage of ATG4

Proteolytic cleavage is an irreversible process mediated by proteases [72]. Autophagy assists in homeostasis by generating energy for programmed cell death [39]. Several autophagy proteins, including ATG5 and Atg6/BECN1, are proteolytic targets of calpain and caspases [73,74]. All four ATG4 paralogs have a central C54 peptidase domain and N- and C-termini for substrate recognition or other regulatory properties [21]. Thus, ATG4s are proteases and are targets of other proteases. Incubation with recombinant caspase-3 reduced full-length ATG4A, ATG4B, and ATG4C in a concentration-dependent manner, and the cleaved ATG4 fragment was detected by Western blotting [39]. The pairwise sequence of ATG4D identified DEVD^63^K, a canonical caspase sequence. This sequence is also conserved in ATG4C, but not in ATG4A or ATG4B. Caspase-3 can cleave ATG4D, but not a ATG4D DEVA^63^K mutant. Furthermore, caspase-3-dependent cleavage of ATG4D enhances delipidation and the activity of its main substrate, GABARAP-L1. The caspase-cleavage-mimicking mutant, ΔN63 Atg4D, further enhanced GAPARAP-L1 delipidation in cells, while ATG4D depletion decreased GAPARAP-L1 processing and autophagosome formation. The overexpression of cleaved ATG4D localized to mitochondria affects the mitochondrial structure and reduces the mitochondrial cristae. This process results in increased ROS production and mitochondrial clearance (mitophagy) [19,21]. Nonetheless, the role of ATG4B cleavage in autophagy and cell death requires further investigation.

## 4. Perspective

Our understanding of autophagy has improved in recent years. Autophagic regulation is critical for maintaining cellular homeostasis under various stress conditions. Many ATG proteins are regulated through post-translational modifications, affecting their ability to modulate autophagy under various stress conditions. ATG4 plays a prominent role in autophagy regulation because it participates in the cleavage of both pro-ATG8 and ATG8-II, two essential steps in autophagosome formation and recycling. However, ATG4 is regulated via multiple modifications. Elucidating how these different modifications interact and how regulation varies in different tissues and under different physiological or pathological conditions is required. As *autophagy* plays an *essential* role in maintaining *homeostasis*, dysregulation of ATG4 is associated with multiple human diseases, including cancer. Understanding the complex post-translational modifications of ATG4 will facilitate the development of a therapeutic strategy for autophagy-associated diseases.

## Figures and Tables

**Table 1:** Post-translational modifications of autophagy-related 4 (ATG4) protein.

Target	Modification	Regulator	Site (* Predicted Site)	Consequence	Cell Type/Model	Reference (PMID)
**ATG4A**	Oxidation	ROS (H_2_O_2_)	C81	Activation	CHO cell	[38] (17347651)
	Cleavage	Caspase-3	EEFD^368^	-	HeLa cells/ In vitro assay	[39] (21121091)
			LEED^392^	-		
		Calpain 1	EKSK^44^LL	-		
			WILG^33^KQ	-		
**ATG4B**	Phosphorylation	-	S383, S392	Activation	HEK293T, MEF cells	[40] (26378241)
		MST4	S383	Activation	Glioma stem-like cells	[35] (29232556)
		AKT1	S34	non-canonical function	HepG2 cells	[41] (29165041)
		AKT2	S121, S262	Activation	HEK293T cells	[42] (30443548)
		PRKP	S34	Activation	HEK293T cells	[43] (33607258)
		ULK1	S316	Inhibition	MEF, HeLa, HEK293T cells	[44] (28821708)
	Dephosphorylation	PP2A	S316	Activation	HEK293T cells	[44] (28821708)
	O-GlcNAcylation	O-GlcNAc transferase	-	Activation	SH-SY5Y cells	[45] (27527864)
	Oxidation	ROS (H_2_O_2_)	C292, C361	Inhibition	HEK293, HeLa cells	[46] (31880198)
	S-Nitrosylation	NO	C189, C292	Inhibition	SH-SY5Y/ hippocampus	[47] (28633005)
	Ubiquitination	RNF5	-	Inhibition	MEF cells	[48] (23093945)
	Cleavage	Caspase-3	EFED^19^	-	HeLa cells/ In vitro assay	[39] (21121091)
			LTYD^9^	-		
		Calpain 1	ERLE^378^RF	-		
**ATG4C**	Cleavage	Caspase-3	DEVD^10^	-	HeLa cells/ In vitro assay	[39] (21121091)
		Calpain 1	KKFT^158^AS	-		
			ASLS^166^GE	-		
**ATG4D**	Cleavage	Caspase-3	DEVD^63^K	Activation	HeLa, A431, HEK293 cells	[21] (19549685)
					HeLa cells	[19] (22441018)
